# Nanoemulsion: an advanced mode of drug delivery system

**DOI:** 10.1007/s13205-014-0214-0

**Published:** 2014-04-08

**Authors:** Manjit Jaiswal, Rupesh Dudhe, P. K. Sharma

**Affiliations:** Department of Pharmacy, School of Medical and Allied Sciences, Galgotias University, G. B. Nagar, Uttar Pradesh India

**Keywords:** Nanoemulsion, Drug delivery, Emulgents, High-pressure homogenization

## Abstract

An advanced mode of drug delivery system has been developed to overcome the major drawbacks associated with conventional drug delivery systems. This review gives a detailed idea about a nanoemulsion system. Nanoemulsions are nano-sized emulsions, which are manufactured for improving the delivery of active pharmaceutical ingredients. These are the thermodynamically stable isotropic system in which two immiscible liquids are mixed to form a single phase by means of an emulsifying agent, i.e., surfactant and co-surfactant. The droplet size of nanoemulsion falls typically in the range 20–200 nm. The main difference between emulsion and nanoemulsion lies in the size and shape of particles dispersed in the continuous phase. In this review, the attention is focused to give a basic idea about its formulation, method of preparation, characterization techniques, evaluation parameters, and various applications of nanoemulsion.

## Introduction

Nanoemulsions are a colloidal particulate system in the submicron size range acting as carriers of drug molecules. Their size varies from 10 to 1,000 nm. These carriers are solid spheres and their surface is amorphous and lipophilic with a negative charge. Magnetic nanoparticles can be used to enhance site specificity. As a drug delivery system they enhance the therapeutic efficacy of the drug and minimize adverse effect and toxic reactions. Major application includes treatment of infection of the reticuloendothelial system (RES), enzyme replacement therapy in the liver, treatment of cancer, and vaccination. An emulsion is a biphasic system in which one phase is intimately dispersed in the other phase in the form of minute droplets ranging in diameter from 0.1 to 100 μm. It is a thermodynamically unstable system, which can be stabilized by the presence of an emulsifying agent (emulgent or emulsifier). The dispersed phase is also known as internal phase or the discontinuous phase while the outer phase is called dispersion medium, external phase or continuous phase. The emulsifying agent is also known as intermediate or interphase. The term ‘nanoemulsion’ also refers to a miniemulsion which is fine oil/water or water/oil dispersion stabilized by an interfacial film of surfactant molecule having droplet size range 20–600 nm. Because of small size, nanoemulsions are transparent. There are three types of nanoemulsion which can be formed: (a) oil in water nanoemulsion in which oil is dispersed in the continuous aqueous phase, (b) water in oil nanoemulsion in which water droplets are dispersed in continuous oil phase, and (c) bi-continuous nanoemulsions.

## Advantages of nanoemulsion

- It may be used as substitute for liposomes and vesicles (Bouchemal et al. 2004a, b).
- It improves the bioavailability of drug (Kim et al. 2001; Wagner et al. 1996).
- It is non-toxic and non-irritant in nature.
- It has improved physical stability.
- Nanoemulsions have small-sized droplets having greater surface area providing greater absorption.
- It can be formulated in variety of formulations such as foams, creams, liquids, and sprays.
- It provides better uptake of oil-soluble supplements in cell culture technology.
- It helps to solubilize lipophilic drug.
- Helpful in taste masking.
- Less amount of energy is required.

## Components of nanoemulsion

The main components of nanoemulsion are oil, emulsifying agents, and aqueous phases (Gasco et al. 1991; Kriwet and Müller-Goymann 1995; Trotta 1999). Oils can be of any type like castor oil, corn oil, coconut oil, evening primrose oil, linseed oil, mineral oil, olive oil, peanut oil, etc. A mixture of oil and water may yield a crude temporary emulsion, which upon standing, will separate in two distinct phases due to the coalescence of the dispersed globules. Emulgents or emulsifying agents can impart stability to such systems. Emulgents are broadly classified as surfactants like spans and tweens, hydrophilic colloids such as acacia and finely divided solids, e.g., bentonite and veegum. An emulgent, in addition to its emulsifying properties, should be nontoxic and its taste, odour and chemical stability should be compatible with the product. Some of the desirable properties of an emulgent are: (1) it should be able to reduce the surface tension to below 10 dynes/cm, (2) it should be adsorbed rapidly around dispersed phase globule to form a complete and coherent film to prevent coalescence, (3) it should help in building up an adequate zeta potential and viscosity in the system so as to impart optimum stability, and (4) it should be effective in a fairly low concentration. Emulgents form monomolecular, multimolecular or particulate films around the dispersed globules (Sharma and Jain 1985).

### Monomolecular films

Surfactant type of emulgents stabilizes a nanoemulsion by forming a monolayer of adsorbed molecules or ions at the interface reducing interfacial tension. In modern day practice, combination of emulgents is preferred over single emulgent. The combination consists of a predominantly hydrophilic emulgent in the aqueous phase and a hydrophobic agent in the oily phase to form a complex film at the interface.

### Multimolecular films

Hydrated lyophilic colloids form multimolecular films around globules of dispersed oil. Hydrated colloids do not cause any appreciable lowering of surface tension and their ability to form strong, coherent multimolecular films. Their tendency to increase the viscosity of the continuous phase enhances the stability of emulsion.

### Solid particulate films

The emulgents forming particulate films are small solid particles that are wetted to some degree by both aqueous and non-aqueous liquid phases. They are concentrated at the interface where they produce a film around the dispersed globules thus preventing coalescence.

## Formulation aspects and method of preparation of nanoemulsion

Formulation of nanoemulsion includes active drug, additive and emulsifier. The various methods for the preparation of nanoemulsion include two methods: (a) high-energy emulsification and (b) low-energy emulsification. The high-energy emulsification method includes high-energy stirring, ultrasonic emulsification, high-pressure homogenization, microfluidization, and membrane emulsification (Tiwari and Amiji 2006; Perdiguer et al. 1997; Banker et al. 2002). The low-energy emulsification method includes phase inversion temperature, emulsion inversion point, and spontaneous emulsification (Ahuja et al. 2008). Using a combined method, which includes the high-energy and low-energy emulsification, it is possible to prepare reverse nanoemulsion in a highly viscous system.

## Ultrasonic emulsification

Ultrasonic emulsification is very efficient in reducing droplet size. In ultrasonic emulsification, the energy is provided through sonotrodes called as sonicator probe. It contains piezoelectric quartz crystal which can expand and contract in response to alternating electric voltage. As the tip of sonicator contacts the liquid, it produces mechanical vibration and cavitation occurs. Cavitation is the formation and collapse of vapour cavities in liquid. Thus, ultrasound can be directly used to produce emulsion; it is mainly used in laboratories where emulsion droplet size as low as 0.2 micrometer can be obtained.

## High-pressure homogenization

The preparation of nanoemulsion requires high-pressure homogenization. This technique makes use of high-pressure homogenizer/piston homogenizer to produce nanoemulsion of extremely low particle size (up to 1 nm) (Asua 2002; Anton et al. 2008).

## Microfluidization

Microfluidization is a patented mixing technology, which makes use of a device called microfluidizer. This device uses high pressure which forces the drug product through the interaction chamber resulting in a very fine particle of submicron range. The process is repeated several times to obtain a desired particle size to produce uniform nanoemulsion.

## Phase inversion temperature

This method involves change in phase by applying a higher temperature to a microemulsion (El-Aasser et al. 1986; Pouton 1997).

## Spontaneous emulsification

It involves three steps: (a) preparation of homogeneous organic solution consisting of oil and lipophilic surfactant in water miscible solvent and hydrophilic surfactant, (b) the organic phase is injected in aqueous phase under continuous magnetic stirring, o/w emulsion is formed, and (c) the aqueous phase is removed by evaporation under reduced pressure (Solans et al. 2005; Tadros et al. 2004).

## Factors to be considered during preparation of nanoemulsion

1. Surfactant must be selected carefully such that an ultralow interfacial tension may be achieved which is a primary requirement to produce nanoemulsion.
2. Concentration of surfactant must be high enough to stabilize the microdroplets to produce nanoemulsion.
3. The surfactant must be flexible or fluid enough to promote the formation of nanoemulsion.

## Characterization of nanoemulsion

A stable nanoemulsion is characterized by the absence of the internal phase, absence of creaming, absence of deterioration by microorganisms, and maintenance of elegance in respect of appearance, colour, odour and consistency (Sharma and Jain 1985). Hence the instability of emulsion can be classified as follows:

### Flocculation and creaming

Flocculation consists of the joining together of globules to form large clumps or floccules, which rise or settle in the emulsion more rapidly than the individual globules. The rising up or settling down of dispersed globules to give a concentrated layer is known as creaming. Thus flocculation leads to creaming.

### Cracking

Cracking of an emulsion refers to separation of the dispersed phase as a layer. Whereas a creamed emulsion may be reconstituted by shaking or agitation, a cracked emulsion cannot be corrected. Cracking represents permanent instability. Cracking of the emulsion may be due to: (1) addition of an emulgent of opposite nature, (2) decomposition or precipitation of emulgent, (3) addition of a common solvent in which both oily and aqueous phases are miscible, (4) extremes of temperature, (5) microorganisms, (6) creaming.

### Miscellaneous instability

Emulsions may deteriorate if stored under extremely high or low temperature or in the presence of light. Hence emulsions are usually packed in air-tight, coloured containers and stored at moderate temperature.

### Phase inversion

It is the change in the type of emulsion from o/w to w/o and vice versa. It is the physical process. Phase inversion may be brought about by varying the phase volume ratio, addition of electrolytes, and temperature changes.

## Evaluation parameters of nanoemulsion

### Droplet size analysis

Droplet size analysis of nanoemulsion is measured by a diffusion method using a light-scattering, particle size-analyzer counter, LS 230. It is also measured by correlation spectroscopy that analyzes the fluctuation in scattering of light due to Brownian motion. Droplet size analysis of nanoemulsion can also be performed by transmission electron microscopy (TEM) (Bouchemal et al. 2004a, b; Alka et al. 2007; Farhan et al. 2008).

### Viscosity determination

The viscosity of nanoemulsion is measured by using Brookfield-type rotary viscometer at different shear rates at different temperatures.

### Dilution test

Dilution of a nanoemulsion either with oil or with water can reveal this type. The test is based on the fact that more of the continuous phase can be added into a nanoemulsion without causing the problem of its stability. Thus, an o/w nanoemulsion can be diluted with water and a w/o nanoemulsion can be diluted with oil.

### Drug content

Preweighed nanoemulsion is extracted by dissolving in a suitable solvent, extract is analyzed by spectrophotometer or HPLC against standard solution of drug (Singh and Vingkar 2008; Chen et al. 2008).

### Polydispersity

It indicates the uniformity of droplet size in nanoemulsion. The higher the value of polydispersity, lower will be uniformity of droplet size of nanoemulsion. It can be defined as the ratio of standard deviation to mean droplet size. It is measured by a spectrophotometer.

### Dye test

If a water-soluble dye is added in an o/w nanoemulsion the nanoemulsion takes up the colour uniformly. Conversely, if the emulsion is w/o type and the dye being soluble in water, the emulsion takes up the colour only in the dispersed phase and the emulsion is not uniformly coloured. This can be revealed immediately by microscopic examination of the emulsion.

### Refractive index

Refractive index of nanoemulsion is measured by Abbes refractometer.

### pH

The pH of nanoemulsion can be measured by pH meter.

### Zeta potential

Zeta potential is measured by an instrument known as Zeta PALS. It is used to measure the charge on the surface of droplet in nanoemulsion (Erol and Hans-Hubert 2005).

### Fluorescence test

Many oils exhibit fluorescence when exposed to UV light. When a w/o nanoemulsion is exposed to a fluorescence light under a microscope, the entire field fluoresces. If the fluorescence is spotty, the nanoemulsion of o/w type.

### Percentage transmittance

Percentage transmittance of nanoemulsion is measured by a UV-visible spectrophotometer.

### Conductance measurement

The conductance of nanoemulsion is measured by a conductometer. In this test a pair of electrodes connected to a lamp and an electric source is dipped into an emulsion. If the emulsion is o/w type, water conducts the current and lamp gets lit due to passage of current between the electrodes. The lamp does not glow when the emulsion is w/o: oil being in external phase does not conduct the current.

### Filter paper test

This test is based on the fact that an o/w nanoemulsion will spread out rapidly when dropped onto filter paper. In contrast, a w/o nanoemulsion will migrate only slowly. This method should not be used for highly viscous creams (Sharma and Jain 1985).

## Conclusion

Nanoemulsions are widely used in pharmaceutical systems. Nanoemulsion formulation offers several advantages such as delivery of drugs, biological or diagnostic agents. The most important application of nanoemulsion is for masking the disagreeable taste of oily liquids. Nanoemulsion may also protect the drugs, which are susceptible to hydrolysis and oxidation. Nowadays, nanoemulsions are used for targeted drug delivery of various anticancer drugs, photo sensitizers or therapeutic agents. Nanoemulsion can also provide prolonged action of the medicaments. Overall all nanoemulsion formulation may be considered as effective, safe and with increased bioavailability. It is expected that further research and development will be carried out in the future regarding nanoemulsion.
